# Flow-through stable isotope probing (Flow-SIP) minimizes cross-feeding in complex microbial communities

**DOI:** 10.1038/s41396-020-00761-5

**Published:** 2020-09-02

**Authors:** Maria Mooshammer, Katharina Kitzinger, Arno Schintlmeister, Soeren Ahmerkamp, Jeppe Lund Nielsen, Per Halkjær Nielsen, Michael Wagner

**Affiliations:** 1 Division of Microbial Ecology, Centre for Microbiology and Environmental Systems Science, University of Vienna, Vienna, Austria; 2 Max Planck Institute for Marine Microbiology, Bremen, Germany; 3 Large-Instrument Facility for Environmental and Isotope Mass Spectrometry, Centre for Microbiology and Environmental Systems Science, University of Vienna, Vienna, Austria; 4 MARUM–Center for Marine Environmental Sciences & Department of Geosciences, University of Bremen, Bremen, Germany; 5 Department of Chemistry and Bioscience, Aalborg University, Aalborg, Denmark

**Keywords:** Microbiology, Biological techniques, Metabolism, Biogeochemistry, Microbial ecology

## Abstract

Stable isotope probing (SIP) is a key tool for identifying the microorganisms catalyzing the turnover of specific substrates in the environment and to quantify their relative contributions to biogeochemical processes. However, SIP-based studies are subject to the uncertainties posed by cross-feeding, where microorganisms release isotopically labeled products, which are then used by other microorganisms, instead of incorporating the added tracer directly. Here, we introduce a SIP approach that has the potential to strongly reduce cross-feeding in complex microbial communities. In this approach, the microbial cells are exposed on a membrane filter to a continuous flow of medium containing isotopically labeled substrate. Thereby, metabolites and degradation products are constantly removed, preventing consumption of these secondary substrates. A nanoSIMS-based proof-of-concept experiment using nitrifiers in activated sludge and ^13^C-bicarbonate as an activity tracer showed that Flow-SIP significantly reduces cross-feeding and thus allows distinguishing primary consumers from other members of microbial food webs.

Stable isotope probing (SIP) is widely applied to link specific microbial populations to metabolic processes in the environment and has greatly advanced our understanding of the role of microorganisms in biogeochemical cycling. SIP relies on tracing the incorporation of specific isotopically labeled substrates (e.g., ^13^C, ^15^N, ^18^O, ^2^H) into cellular biomarkers or bulk cellular biomass [e.g., 1–4]. SIP is considered a robust technique to identify microbial populations that assimilate a labeled substrate of interest in complex environmental communities. However, cross-feeding can occur when isotopically labeled metabolites are released from a primary consumer and then used by other microorganisms, which subsequently also become isotopically labeled. Likewise, when ^13^C-bicarbonate and unlabeled substrate are supplied to assess the activity of specific chemolithoautotrophs [e.g., 5–7], undesired ^13^C-incorporation can occur due to cross-feeding between chemolithoautotrophs whose activity depends on each other, for example in nitrifiers, where ammonia oxidizers provide nitrite oxidizers with their substrate, nitrite. The uncertainties associated with cross-feeding in SIP studies increase as the incubation time of microbial communities increases. While this phenomenon can be used to study microbial interactions and trophic networks [8–11], cross-feeding can lead to erroneous identification of organisms that are not directly responsible for the process of interest, but are rather connected to primary consumers via a microbial food web [2, 10, 12, 13].

We developed an approach that significantly reduces the effect of cross-feeding in SIP studies. For this purpose, a thin layer of microbial cells is placed on a membrane filter, and isotopically labeled substrate is supplied at a fixed concentration by continuous flow, which constantly removes released metabolites and degradation products of primary substrate consumers. While previous SIP studies have employed a continuous flow of medium or substrate [e.g., 6, 14–16], in these studies, cross-feeding still occurred, as large amounts of biomass were placed in a 3D space, which allowed for the exchange of metabolites. Here, we present a proof-of-concept experiment with a nitrifying activated sludge microbial community, which converts ammonia to nitrite by the activity of ammonia-oxidizing bacteria (AOB), and subsequently oxidizes nitrite to nitrate by nitrite-oxidizing bacteria (NOB). In our experiments, the carbon source for both groups of autotrophic nitrifiers (the sludge contained no comammox bacteria [17, 18]) was isotopically labeled inorganic carbon (^13^C–NaHCO_3_) and, as the sole electron donor, unlabeled ammonium was provided.

In the flow-through approach, AOB, but not NOB, should be ^13^C-labeled because the substrate for NOB (nitrite), produced by AOB is continuously removed and thus the NOB should remain metabolically inactive (Fig. 1). In addition to a regular batch incubation, we included a control incubation, where the flow-through was recirculated to determine the impact of the experimental setup (continuous medium flow and retainment of biomass on a membrane filter) in Flow-SIP on the activity of the bacterial cells (in particular on the NOB as their autotrophic activity is used as a read out for cross-feeding in our experiments) in comparison to the batch experiment. Cross-feeding is expected to occur in both recirculated and batch control incubations, where nitrite is not removed and thus both AOB and NOB conserve energy to fix ^13^C–CO_2_. After the experiments, fluorescence in situ hybridization (FISH) with rRNA-targeted oligonucleotide probes was used to identify AOB and NOB and combined with nanoscale secondary ion mass spectrometry (nanoSIMS) to quantify ^13^C-assimilation at the single-cell level for all setups.

**Fig. 1:**
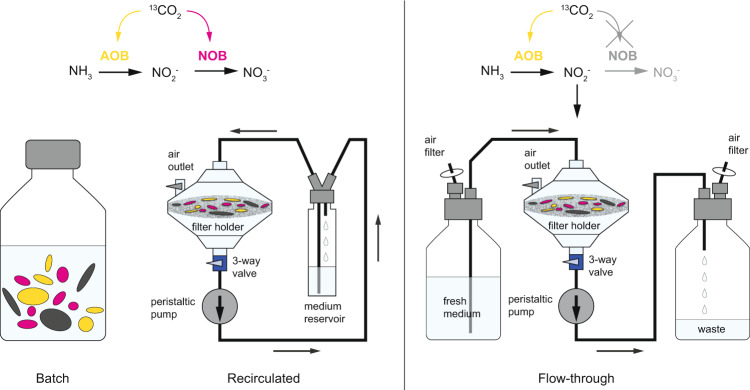
Schematic representation of the experimental setup: (left) batch, (center) recirculated, and (right) flow-through incubation. In all incubations, the carbon source for both autotrophic nitrifiers (AOB, yellow and NOB, magenta) was isotopically labeled (i.e., CO_2_ as ^13^C–NaHCO_3_) and ammonia was provided as the only external energy source (as NH_4_Cl). Cross-feeding is expected to occur in the batch and recirculated approaches, where NOB consume nitrite produced via ammonia oxidation by AOB and thus both AOB and NOB incorporate ^13^C–CO_2_. In the flow-through approach, only AOB are expected to be ^13^C-labeled, as cross-feeding should be eliminated by the continuous removal of nitrite. Other, non-nitrifier cells are indicated in gray.

For these experiments, activated sludge from a Danish municipal wastewater treatment plant was initially treated by sonication to disrupt large flocs. Cells were then either placed on a membrane filter for flow-through incubation and the recirculated control experiment, or incubated in a conventional batch experiment. All experiments were set up using the same amount of biomass, and the ratio of biomass to medium volume was the same in batch and recirculated control experiments. Incubations were done using mineral medium containing 250 µM NH_4_Cl and 2 mM ^13^C–NaHCO_3_ for 24 h. Medium flow was maintained at a rate of 26 ml h^−1^. We did not select a higher flow rate in order to avoid excessive stress by the medium flow on the microbial cells and to minimize the required amounts of media containing isotopically labeled bicarbonate. Furthermore, modeling nitrite advection and diffusion at different flow rates showed that, for example, a tenfold higher flow rate would only marginally reduce nitrite concentrations surrounding the AOB colonies (Fig. S1). In contrast, in a purely diffusive system without continuous flow, our model showed that nitrite would accumulate to significantly higher concentrations around single AOB colonies (Fig. S2). For example, after 24 h, ~23 µM nitrite would accumulate at a distance of 0–100 µm (with no significant decrease over distance) around an AOB colony of 50 cells, which is 230- to 9200-fold higher (depending on the distance to the colony) than modeled nitrite concentrations at the flow rate used in our experiments. Most inorganic metabolites that can directly be taken up into cells behave similar as nitrite in a diffusive system, i.e., they have a similar diffusion coefficient. Larger molecules tend to have an even smaller diffusion coefficient, which would lead to slower diffusion, and thus even more efficient metabolite removal when a medium flow is applied.

We monitored nitrification activity via concentration measurements of ammonium, nitrite and nitrate (Fig. S3) and conducted nanoSIMS analyses (Fig. 2) for two successive experiments using sludge collected from the same treatment plant on different days as replication of experimental results (referred to as E1 and E2). Additionally, we confirmed the reproducibility of the method with two further experiments, where nitrification activity was followed (Fig. S4). Details on the experimental setup are given in Fig. 1 and the Supplementary Text.

**Fig. 2:**
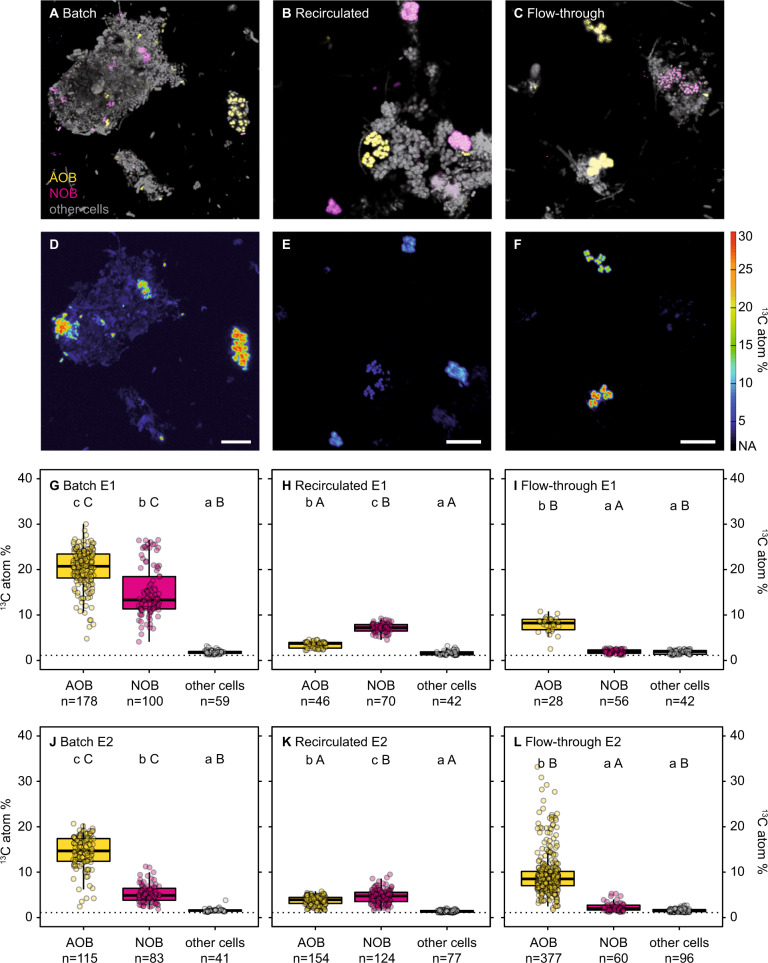
Single cell isotope probing of nitrifying activated sludge in batch, recirculated, and flow-through incubations. **a**–**c** Show representative FISH images of E2 (AOB in yellow; NOB in magenta; other cells counterstained by DAPI in gray) of batch, recirculated, and flow-through incubations, respectively, and **d**–**f** show the corresponding nanoSIMS images. Scale bar is 10 µm in all images. **g**–**l** Show ^13^C labeling of AOB, NOB, and other cells quantified by nanoSIMS at the single-cell level for E1 (**g**–**i**) and E2 (**j**–**l**) in batch, recirculated, and flow-through incubations, respectively. We used FISH probe sets targeting AOB (*Nitrosomonas oligotropha* cluster (Cl6a192), *Nitrosomonas eutropha/europea/urea* cluster (NEU)) and NOB (*Nitrotoga* (Ntoga122), *Nitrospira* Lineage 1 (Ntspa1431), and *Nitrospira* Lineage 2 (Ntspa1151)), respectively, for differential staining of the two nitrifier groups. In (**g**–**l**), dashed lines give the ^13^C natural abundance values of the filter surface. The number of cells analyzed per group is indicated below each boxplot. For each experiment, lower case letters indicate significant difference in ^13^C labeling between groups (AOB, NOB, other cells) within an incubation type and upper case letters indicate significant difference between incubation types for a given group (Kruskal–Wallis test followed by Dunn’s test; Statistics are given in Table S2).﻿ Boxplots depict the 25–75% quantile range, with the center line depicting the median (50% quantile) and whiskers encompass data points within 1.5× the interquartile range.

In recirculated and batch control incubations, the consumption of ammonium, production of nitrite and nitrate (Fig. S3), and single cell ^13^C-incorporation (Fig. 2) indicated that both AOB and NOB were active. However, nitrification activity (i.e., nitrite and nitrate production) in recirculated incubations were reduced by 57% (E1) and 83% (E2) compared to batch incubations (Fig. S3). The reduced ammonia oxidation activity in the recirculated incubations compared to the batch incubations was also reflected by a 73–82% lower ^13^C-incorporation in AOB cells in the former incubations (Fig. 2, median AOB ^13^C-enrichment in recirculated setup was 3.7 and 3.9 ^13^C-atom%, in batch incubations 20.7 and 14.7 ^13^C-atom% for E1 and E2, respectively). AOB in the flow-through incubations also showed lower ^13^C-enrichment levels (8.2 and 8.5 atom% for E1 and E2, respectively) compared to batch incubations but higher enrichment than in the recirculated incubations. The lower enrichment of AOB in the recirculated compared to the flow-through incubations might be due to an accumulation of compounds leaching from the used tubing (PharMed^®^ Ismaprene, Table S1), which may negatively affect AOB. Indeed, nitrifiers have previously been reported to be sensitive to various organic compounds [19, 20]. Use of different rubber tubing or replacing rubber tubing by glass might alleviate these effects. AOB ^13^C-enrichment was highest in batch incubations, which could be due to both the lack of stress from the continuous medium flow and the observed reaggregation of the sonicated activated sludge into larger flocs—reminiscent of native activated sludge flocs.

As expected, NOB were ^13^C-enriched in both the batch (13.3 and 4.9 atom% for E1 and E2, respectively) and recirculated incubations (7.2 and 4.7 atom% for E1 and E2, respectively). In contrast, as intended, the flow-through setup resulted in a substantial reduction in ^13^C-enrichment of NOB (2.0 atom% for both E1 and E2, respectively; with consistently low ^13^C-enrichment in all NOB cells measured). This demonstrates that Flow-SIP efficiently removed the secondary substrate nitrite released by the AOB primary substrate consumers, thereby strongly limiting cross-feeding between AOB and NOB. The low ^13^C-enrichment of NOB in the flow-through incubations was statistically not significantly different to the ^13^C-enrichment of non-nitrifier cells (Table S2). It is unlikely that this low background ^13^C-enrichment was due to ^13^C-bicarbonate adsorption, as all samples were treated with acid before nanoSIMS analysis. It is, however, possible that at least some of the observed ^13^C-enrichment in NOB and other bacteria is due to anaplerotic reactions leading to C-fixation by background cellular activity rather than substrate-induced autotrophic C-fixation [e.g., 21, 22]. Transfer of ^13^C-labeled metabolites from the autotrophic nitrifiers to non-nitrifier cells was negligible in all incubations, including batch and recirculated incubations (Fig. 2), which was likely due to the short incubation time (24 h). In contrast, other SIP studies using incubation times of several days reported significant C-isotope transfer from nitrifiers to non-nitrifiers [11, 23].

Our results demonstrate that Flow-SIP is a promising approach to significantly reduce cross-feeding in complex microbial communities and can be even successfully applied to highly aggregated samples like activated sludge flocs when they are dispersed prior to the experiment. Flow-SIP and conventional SIP are complementary to each other in the analysis of such aggregated or biofilm communities. In such systems, Flow-SIP enables microbial ecologists studying microbial physiologies with drastically reduced cross-feeding, but destroys the spatial arrangement of cells, while conventional SIP retains the 3D architecture, but its results are strongly influenced by cross-feeding. We expect that Flow-SIP is ideally suited for oligotrophic fresh- or seawater samples, which predominantly harbor planktonic cells or small aggregates, and thus do not require any sonication pretreatment before incubation. Furthermore, for such samples, tracer can be directly added to sterile filtered water without the need for using artificial medium, as used for the presented proof-of-principle experiments.

Flow-SIP may, after upscaling to label more biomass, also be used in combination with DNA-, RNA- or protein-SIP, which should in comparison to conventional SIP, where cross-feeding is not inhibited, allow microbial ecologists to more precisely identify both previously known and yet unknown primary consumers of a supplied substrate. For example, Flow-SIP with ^13^C-bicarbonate and unlabeled ammonium would allow distinguishing comammox organisms from canonical NOB, as comammox but not the canonical NOB would be active under these conditions together with the canonical ammonia oxidizers. In addition, Flow-SIP has the potential to study direct use of chemically unstable substrates by microorganisms, by distinguishing it from microbial consumption of their chemically formed decomposition products. For example, cyanate, which abiotically decays relatively fast to ammonium and carbon dioxide [24, 25], has previously been shown to serve as energy and nitrogen source for ammonia-oxidizing archaea [25, 26]. Using Flow-SIP, cyanate could be constantly supplied, thereby strongly reducing abiotic decay. At the same time, any abiotically formed ammonium (and ammonium produced by other organisms) would be constantly removed, which should allow identifying ammonia-oxidizing microorganisms that directly use cyanate as a substrate. Furthermore, the presented approach may be coupled to fluorescence-based activity markers, where a substrate of interest and bioorthogonal noncanonical amino acids are supplied and, subsequently, translationally active cells are visualized on an epifluorescence microscope (BONCAT) [27]. In conclusion, Flow-SIP expands the toolbox of microbial ecologists interested in structure–function analyses of microbial communities and will contribute to a more precise understanding of the ecophysiology of bacteria and archaea catalyzing key processes in their natural environments.

## Supplementary information

Supplementary Information
