# Supplementary material for: Flow-through stable isotope probing (Flow-SIP) minimizes cross-feeding in complex microbial communities
Source: ISME J. 2020 Sep 2;15(1):348–53. doi: 10.1038/s41396-020-00761-5 (PMC7852690; doi:10.1038/s41396-020-00761-5)
Supplement: Supplementary file 1 — Supplementary Information [file 41396_2020_761_MOESM1_ESM.pdf]

Supplementary information: Flow-through stable isotope probing (Flow-SIP) minimizes cross-feeding in complex microbial communities

Supplementary text

Materials and experimental setup

Schemes of the incubation setup of recirculated and flow-through approaches are shown in Figure 1. For the flow-through approach, the screw caps of the medium reservoir and the waste collection bottle were equipped with two ports, one for the tubing inlet and one for sterile pressure equalization using a membrane filter (0.2 µm). For the recirculated approach, a small bottle was used as medium reservoir with two ports, from which medium was withdrawn and recirculated back, respectively. For both approaches, the medium reservoir was connected to the top of the filter holder (stainless steel, 47 mm, Sartorius) and a peristaltic pump was placed after the filter holder. To open and close the filter holder as well as to remove aliquots of the medium for chemical analysis during the incubation, a three-way valve was connected to the bottom of the filter holder. Material used for both recirculated and flow-through approaches are given in Table S1. All material except for the three-way-valves was sterilized by autoclaving, three-way valves were sterilized by rinsing in 70% ethanol and autoclaved distilled water. Mineral medium was prepared according to Palatinszky *et al.* [1], with some modifications: instead of 4 g L^{-1} $CaCO_3$, we used 0.01 g L^{-1} (1 mM, as a calcium source), and, as substrate for autotrophic C-fixation, we added ^{13}C-$NaHCO_3$ (98 atom%) with a final concentration of 2 mM, resulting in a final ^{13}C-labelling percentage of approximately 66 atom%. For batch and recirculated incubations, 20 ml of medium were inoculated. We accounted for the dead volume of the filter holder and tubing (9 ml) and thus only 11 ml medium were added to the medium reservoir of the recirculated approach. In the flow-through incubations, approximately 624 ml of medium were supplied over 24 h (i.e., flow rate of 26 mL h^{-1}).

Activated sludge was sampled from the nitrification basin of the municipal wastewater treatment plant Aalborg West (luftningstanke, Renseanlæg Vest), Denmark, on March 16th and 18th, 2016. Sludge was disrupted by sonication to yield single cells or colonies to reduce diffusive cross-feeding due to the large 3D-structure of the flocs. 20 ml aliquots of sludge (diluted 1:5 in mineral medium) were sonicated on ice for 120 s using 20% power and 1x cycle settings (Bandelin HD2200, probe MS73), and were allowed to settle for 10 min. The top 15 ml of the sludge suspensions, containing smaller flocs or single colonies and cells, were filtered through 20 µm nylon mesh membrane filters (Magna, Fisher Scientific) to remove residual large flocs. To remove residual substrates from the filtrate, cells were pelleted by centrifugation (20 min, 10°C, 4 200 g), supernatant was discarded, and cells were gently resuspended in mineral medium with ^{12}C-$NaHCO_3$ and without ammonium. To avoid the use of storage compounds during the incubation, cells were exposed to a starvation period of approximately 8 - 10 h before start of the incubation. To determine optimal cell density for the incubations, different volumes of sonicated, washed cells were filtered onto 0.2 µm filters, stained by DAPI and inspected by an epifluorescence microscope.

Under sterile conditions, support filters (for E1, glass fibre filters, Advantec, GC50, 47mm, and for E2 nylon membrane filters Magna, Fisher Scientific, 47mm) and membrane filters (0.2µm polycarbonate, Nucleopore Whatman 47mm) were placed into filter holders (backpressure grids were omitted) fitted with a valve at the filter holder outlet. Filter holders were then filled bubble-free with mineral medium and closed off until cells were applied onto the filter membrane. Using syringes, sonicated, starved cells were gently filtered onto the membranes, discarding the flow-through. To ensure even settling of cells on the membrane surface, additional 20 ml mineral medium (with ^{12}C-$NaHCO_3$ and without ammonium) were gently pushed through the filter holder, discarding the flow-through. Shortly before the start of the incubation, the medium in the filter holder was replaced by medium containing ^{13}C-$NaHCO_3$ (but no ammonium). Filter holders were then connected to sterile tubing of the incubation setup (Figure 1). The same amount of biomass as in the recirculated and flow-through incubations were added to the batch incubations. At the start of the incubation, ammonium was added from a stock solution to batch incubation bottles and into the medium reservoir and fresh medium bottle of

the recirculated and flow-through approaches, respectively. Incubations were done in the dark for 24h at *in situ* temperature of the nitrification basin in Aalborg Vest (14°C). Immediately after the setup of the incubation, and after approximately 12, 18 and 24 h, subsamples were collected for concentration measurements of ammonium, nitrite and nitrate. In both flow-through and recirculated incubations, samples were collected at the filter holder outlet, and from batch incubations, bulk samples were collected, and cells were removed by centrifugation. Samples were stored at -20°C until analysis.

At the end of the incubation, filter holders were closed off using the valve at the filter outlet, disconnected from the tubing, and cells on the membrane filter inside the filter holders were fixed with formaldehyde (3% formaldehyde in 1x phosphate buffered saline; PBS). To avoid disturbing the cells' position on the membrane filter, 20 ml formaldehyde solution were gently pushed through the filter holders, thereby replacing medium. Filter holders were then closed using the valve connected at the filter holder outlet and incubated for 30 min at room temperature. Formaldehyde was removed by pushing 20 ml 1x PBS through the filter holder, followed by 20 ml distilled water. After pushing out all liquid, filter holders were disassembled, and membrane filters were air-dried and frozen at -20°C until FISH and nanoSIMS analyses. Batch incubation samples were also filtered on a membrane filter, fixed and stored the same way as the flow-through and recirculated samples.

Nitrification activity was monitored by ammonium consumption, and nitrite and nitrate production. Ammonium, nitrite and nitrate concentrations were quantified after 0, 12, 18 and 24 h by a colorimetric procedure as described in Hood-Nowotny *et al.* [2] and Garcia-Robledo *et al.* [3].

FISH and nanoSIMS analyses

In addition to cells fixed after incubation, bulk activated sludge samples were fixed with formaldehyde as previously described [4]. These samples were used to screen for presence of nitrifier populations by FISH using probes for AOB (probes NEU, [5]; Nso1225, [6]; Nmv/Ncmob [7]; Ncom1025, [8]; Cl6a192, [9]) and NOB (Nit3, [5]; Ntspa1431 and Ntspa1151, [10]; Ntoga122, [11]). We detected AOB populations related to *Nitrosomonas oligotropha* (targeted by probe Cl6a192) and *Nitrosomonas eutropha/europea/urea* (targeted by probe NEU). We further detected *Nitrotoga*-affiliated NOB and

lineage 1 and 2 *Nitrospira* (targeted by probe Ntspa 1431 and 1151, respectively). Previous metagenomic analyses showed that WWTP Aalborg West does not harbor comammox *Nitrospira* [12, 13], whose presence would have confounded the results of our study, as comammox *Nitrospira* are able to oxidize ammonia but are not distinguishable from the canonical lineage 2 *Nitrospira* by FISH. For all combined FISH and nanoSIMS analyses, we used probe mixes for AOB (Cl6a192 and NEU) and NOB (Ntoga122, Ntspa1431 and Ntspa1151).

Before FISH analysis, laser markings were made on membrane filters using a laser microdissection microscope (Leica LMD 7000, Germany). FISH on sections of the incubated filters was done as previously described by Daims [4]. All FISH probes were double labelled with FitC (AOB in E1; NOB in E2), Cy3 (NOB in E1), or Cy5 (AOB in E2) fluorophores [14]. We observed strong non-specific binding of the fluorophores, especially of Cy3 and Cy5, to the membrane filter surface. This non-specific binding was overcome by using CARD-FISH hybridization buffer [15] instead of normal FISH hybridization buffer in hybridizations of E1 samples. Filter sections of E2 were pre-incubated in 1:10 diluted blocking reagent before hybridization [15]. After FISH, cells were counterstained with DAPI before fluorescent images were acquired on a confocal laser scanning microscope (SP7, Leica, Germany, equipped with a white light laser).

For nanoSIMS analyses, selected filter sections were attached to antimony-doped silicon wafer platelets (7.1 x 7.1 x 0.11 mm, Active Business Company, Brunnthal, Germany) by a commercially available superglue (Loctide®, Henkel, Ireland), and coated with AuPd thin films (30 nm nominal thickness) using a sputter coater (K550X Emitech, Quorum Technologies Ltd., Ashford, UK). In the flow-through incubations, a thin, yellow layer of salt crystals was formed on the filter membranes, which strongly reduced the conductivity of the sample surfaces upon sputtering with AuPd. To remove this layer prior to AuPd coating, we tested washing the filter membrane sections from experiment E2, flow-through incubation in either 1N HCl or 1N HNO_3 for 10 min. Both acid treatments successfully removed the yellow layer and rendered the samples sufficiently conductive for nanoSIMS analyses. We subsequently used HCl for washing all other samples. After acid treatment, samples were rinsed with water (Milli-Q, >18.2 MOhm, Millipore) and 70% ethanol. For maintaining comparability and to remove

possible ^{13}C-bicarbonate contamination, samples on filter membranes from batch and recirculated incubations were also washed with 1N HCl in the same way.

NanoSIMS measurements were performed on a NanoSIMS 50L (Cameca, Gennevilliers, France) at the Large-Instrument Facility for Environmental and Isotope Mass Spectrometry at the University of Vienna. Prior to data acquisition, analysis areas were pre-sputtered utilizing a high-intensity, slightly defocused Cs^+ ion beam (400 pA beam current, ~1,5 µm spot size). Data were acquired as multilayer image stacks by sequential scanning of a finely focused Cs^+ primary ion beam (approximately 80 nm probe size at 2 pA beam current) over areas between 36 × 36 and 74 × 74 µm^2 at 512 × 512 pixel image resolution and a primary ion beam dwell time of 5 to 10 ms/(pixel∗cycle). The detectors were positioned to enable parallel detection of $^{12}C_2^-$, $^{12}C^{13}C^-$, $^{12}C^{14}N^-$, $^{31}P^-$ and $^{32}S^-$ secondary ions and the mass spectrometer was tuned to achieve a mass resolving power (MRP) of >9 000 (according to Cameca's definition) for detection of C_2^- and CN^- secondary ions, respectively.

NanoSIMS images were processed using the software WinImage version 2.0.8 (Cameca, France). Prior to stack accumulation, the individual images were aligned to compensate for positional variations arising from primary ion beam and/or sample stage drift. Secondary ion signal intensities were dead time corrected on a per-pixel basis. C isotope composition images displaying the $^{13}C/(^{12}C+^{13}C)$ isotope fraction, designated as ^{13}C atom%, were inferred from the C_2^- secondary ion signal intensity distribution images via per-pixel calculation of $^{13}C^{12}C^-/(2\cdot{}^{12}C_2^-+^{13}C^{12}C^-)$ intensity ratios.

Regions of interest (ROIs), referring to individual cells, were manually defined utilizing the nitrogen-, phosphorus- and sulfur-related secondary ion signal intensity distribution maps as indicators of biomass and the respective FISH image. These ROIs were cross-checked by the topographical/morphological appearance of the sampled areas in secondary electron intensity distribution images that were recorded simultaneously with the secondary ion images. Statistical significance of the difference of the ^{13}C-enrichment between groups within each approach was analyzed by the Kruskal-Wallis test followed by a non-parametric multiple comparison test (Dunn's test), using the R package "dunn.test" [16].

Nitrite diffusion model

In order to determine nitrite distributions surrounding the AOB colonies and to quantify the potential exchange rates with NOB colonies, the flow-condition around a single AOB colony on the Flow-SIP filter was simulated.

The steady-state Navier-Stokes equations are given by:

$$\rho(\boldsymbol{u}\cdot\nabla)\boldsymbol{u} = -\nabla \mathrm{p} + \mu\nabla^2\mathbf{u}$$

With the continuity equation for incompressible fluids:

$$\nabla\cdot\boldsymbol{u} = 0,$$

where ρ is the density, ∇ is the del operator, μ the dynamic viscosity of water, p the pressure and $\boldsymbol{u}$ the velocity vector. The time-dependent advection-diffusion equation is given by:

$$\frac{\partial C}{\partial t} = -\mathrm{D}\nabla^2 C + \boldsymbol{u}\cdot\nabla C,$$

where C is the nitrite concentration and $D = 1.7\cdot 10^{-9}\ \mathrm{m}^2\ \mathrm{s}^{-1}$ is the isotropic diffusion of nitrite. The equations were solved in the COMSOL Multiphysics® software.

Simulating the full 3-dimensional complexity as found on the Flow-SIP filter is numerically expensive and requires precise information about the cell and colony structures. Therefore, certain assumptions were made when generating the model domain. First, it is assumed that the AOB colonies are of spherical shape with a radius that can be estimated from measured volumes. For AOB colonies exceeding 50 cells, it was shown that the colony volume can be empirically described using: $V_{col} = {n_{cell}}^{1.56}e^{-3.28}$, where n_{cell} is the cell number [17]. For colonies with fewer cells no empirical relation exists. Therefore, the volume for 50 cells was linearly scaled down to a theoretical volume of a single

cell multiplied with the cell number: $V_{col} = \frac{50^{0.56}e^{-3.28}}{50} \cdot n_{cell}$, which is valid for 1-50 cells. Finally, within the domain the reactive, spherical AOB colony was centered in a cylinder. To minimize wall-effects, the radius of the cylinder was adjusted to 120 µm, which is more than 20 times the radius of the AOB colony.

To mimic conditions of the experimental setup, a symmetric outer boundary with a constant inlet flow velocity U_0 and a no-slip boundary condition along the colony's surface was assumed. The inflow nitrite-concentration was adjusted to 0 µmol l^{-1}. To simulate the nitrite production of the AOB colonies, a constant normal flux (J_n) was imposed at the surface of the colony, which was calculated based on the cell specific volumetric rates and normalized to the surface area:

$$J_{\mathbf{n}} = \frac{n_{cell} \cdot R_{cell}}{S_{col}}$$

With a cell specific volumetric rate of $R_{cell} = 2.6 \text{ fmol cell}^{-1}\text{h}^{-1}$ [17–20] and a surface area of $S_{col} = 4\pi {r_{col}}^2$. The described approach reduces the decisive parameters to cell numbers and flow velocity. In more than 200 stationary model runs ($\frac{dC}{dt} = 0$), cell numbers were varied between $n_{cell} = 5, 50, 500$ cells and the imposed flow velocity was sequentially increased from U_0 = 0.1 - 100 µm s^{-1} in 1.25 µm s^{-1} steps.

Post-processing was performed in Matlab (Mathworks 2017b). Briefly, nitrite concentrations were extracted along the equator up to a distance of 100 µm of the colony surface. This procedure was repeated for all imposed flow velocities and subsequently interpolated to an equidistant grid. Subsequently, contour lines were extracted.

Additionally, time-dependent nitrite accumulation around AOB colonies without flow ($\mathbf{u} = 0$), i.e. in a diffusion-controlled system, were simulated in the same domain as described above. After a simulation time of 24 hours, nitrite profiles along the equator were extracted for 5, 50 and 500 cells (Figure S2).

Supplementary references

1. Palatinszky M, Herbold C, Jehmlich N, Pogoda M, Han P, Bergen M Von, et al. Cyanate as an energy source for nitrifiers. *Nature* 2015; **524**: 105–108.
2. Hood-Nowotny R, Umana NH-N, Inselbacher E, Oswald- Lachouani P, Wanek W. Alternative Methods for Measuring Inorganic, Organic, and Total Dissolved Nitrogen in Soil. *Soil Sci Soc Am J* 2010; **74**: 1018.
3. García-Robledo E, Corzo A, Papaspyrou S. A fast and direct spectrophotometric method for the sequential determination of nitrate and nitrite at low concentrations in small volumes. *Mar Chem* 2014; **162**: 30–36.
4. Daims H. Use of fluorescence *in situ* hybridization and the daime image analysis program for the cultivation-independent quantification of microorganisms in environmental and medical samples. *Cold Spring Harb Protoc* 2009; **4**: 1–7.
5. Wagner M, Rath G, Amann R, Koops H-P, Schleifer K-H. *In situ* Identification of Ammonia-oxidizing Bacteria. *Syst Appl Microbiol* 1995; **18**: 251–264.
6. Mobarry B, Wagner M, Urbain V, Rittmann BE, Stahl D a. Phylogenetic probes for analyzing abundance and spatial organization of nitrifying bacteria. *Appl Environ Microbiol* 1996; **62**: 2156–2162.
7. Pommerening-Röser A, Rath G, Koops H-P. Phylogenetic Diversity within the Genus *Nitrosomonas*. *Syst Appl Microbiol* 1996; **19**: 344–351.
8. Juretschko S. Mikrobielle Populationsstruktur und -dynamik in einer nitrifizierenden/denitrifizierenden Belebtschlammanlage. 2000. Technische Universität München.
9. Adamczyk J, Hesselsoe M, Iversen N, Horn M, Lehner A, Nielsen PH, et al. The Isotope Array, a New Tool That Employs Substrate-Mediated Labeling of rRNA for Determination of Microbial Community Structure and Function. *Appl Environ Microbiol* 2003; **69**: 6875–6887.
10. Maixner F, Noguera DRD, Anneser B, Stoecker K, Wegl G, Wagner M, et al. Nitrite concentration

influences the population structure of *Nitrospira*-like bacteria. *Environ Microbiol* 2006; **8**: 1487–1495.

11. Lücker S, Schwarz J, Gruber-Dorninger C, Spieck E, Wagner M, Daims H. *Nitrotoga*-like bacteria are previously unrecognized key nitrite oxidizers in full-scale wastewater treatment plants. *ISME J* 2015; **9**: 708–720.

12. Albertsen M, Hansen LBS, Saunders AM, Nielsen PH, Nielsen KL. A metagenome of a full-scale microbial community carrying out enhanced biological phosphorus removal. *ISME J* 2012; **6**: 1094–1106.

13. Munck C, Albertsen M, Telke A, Ellabaan M, Nielsen PH, Sommer MOA. Limited dissemination of the wastewater treatment plant core resistome. *Nat Commun* 2015; **6**: 8452.

14. Stoecker K, Dorninger C, Daims H, Wagner M. Double labeling of oligonucleotide probes for fluorescence *in situ* hybridization (DOPE-FISH) improves signal intensity and increases rRNA accessibility. *Appl Environ Microbiol* 2010; **76**: 922–6.

15. Pernthaler A, Pernthaler J, Amann R. Sensitive multi-color fluorescence *in situ* hybridization for the identification of environmental microorganisms. In: Kowalchuk G, de Bruijn FJ, Head IM, Akkermans ADL, van Elsas JD (eds). *Molecular Microbial Ecology Manual*, 2nd ed. 2004. Kluwer Academic Publishers, Dordrecht, Boston, London, pp 711–726.

16. Dinno A. dunn.test: Dunn's Test of Multiple Comparisons Using Rank Sums. R package version 1.3.2. http://CRAN.R-project.org/package=dunn.test. 2007.

17. Coskuner G, Ballinger SJ, Davenport RJ, Pickering RL, Solera R, Head IM, et al. Agreement between Theory and Measurement in Quantification of Ammonia-Oxidizing Bacteria. *Appl Environ Microbiol* 2005; **71**: 6325–6334.

18. Laanbroek HJ, Bodelier PLE, Gerards S. Oxygen consumption kinetics of *Nitrosomonas europaea* and *Nitrobacter hamburgensis* grown in mixed continuous cultures at different oxygen concentrations. *Arch Microbiol* 1994; **161**: 156–162.

19. Daims H, Ramsing NB, Schleifer K, Wagner M. Cultivation-Independent, Semiautomatic

Determination of Absolute Bacterial Cell Numbers in Environmental Samples by Fluorescence *In Situ* Hybridization. *Appl Environ Microbiol* 2001; **67**: 5810–5818.

20. Stieglmeier M, Mooshammer M, Kitzler B, Wanek W, Zechmeister-Boltenstern S, Richter A, et al. Aerobic nitrous oxide production through N-nitrosating hybrid formation in ammonia-oxidizing archaea. *ISME J* 2014; **8**: 1135–1146.

Supplementary figures

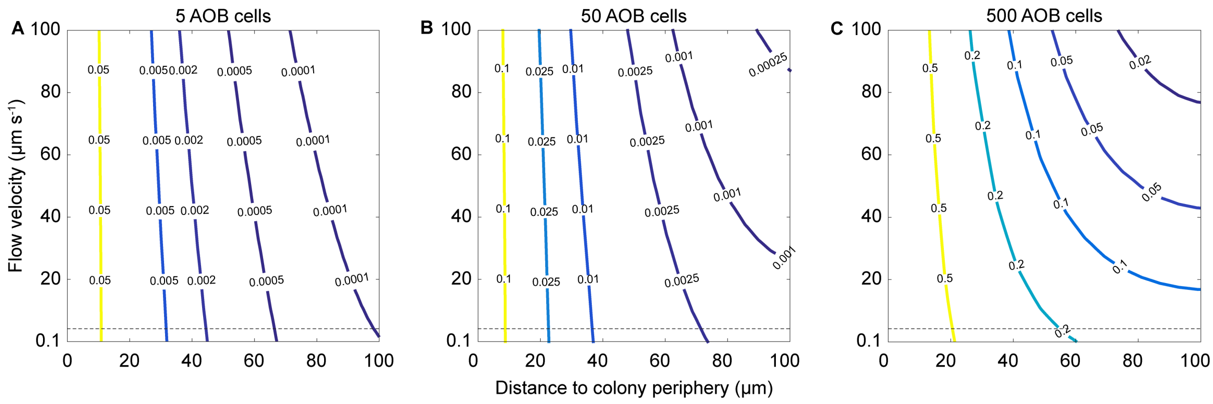

Figure S1. Nitrite diffusion model showing spatial distribution of nitrite concentrations (µM) around AOB colonies on the membrane filter in the flow-through approach, considering different flow velocities from 0.1 – 100 µm s^{-1}. The flow rate of 26 ml h^{-1} used in our experiment corresponds to a flow velocity of 4.2 µm s^{-1} (horizontal dashed grey line). The model was run for three different AOB colony sizes, consisting of either (A) 5, (B) 50 or (C) 500 cells. We analyzed AOB colonies ranging from single cell to approximately 250 cells in our experiments. The distance to the periphery of an AOB colony of 0 – 100 µm corresponds approximately to the distances between AOB and NOB colonies that we have observed. The contour lines represent nitrite concentration (µM).

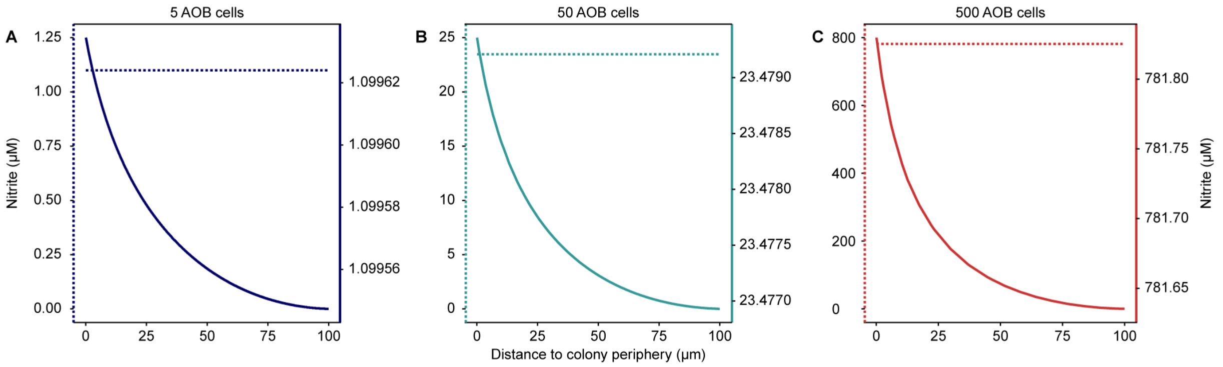

Figure S2. Spatial distribution of nitrite concentrations (µM) in absence of any flow surrounding AOB colonies after 24h of incubation. The model was run for three different AOB colony sizes, consisting of either (A) 5, (B) 50 or (C) 500 cells. Note the different scales on the y-axes – the solid lines (right y-axis) were added to depict very minor changes in nitrite concentrations with distance to the AOB colony peripheries (solid lines). At distances of 0-100 µm, modeled nitrite concentrations around AOB colonies were very stable. Specifically, predicted nitrite concentrations over this distance around AOB colonies consisting of 5 cells were approx. 1.1 µM, around AOB colonies of 50 cells 23 µM, and around AOB colonies of 500 cells 780 µM after 24 h, as depicted by the dotted lines.

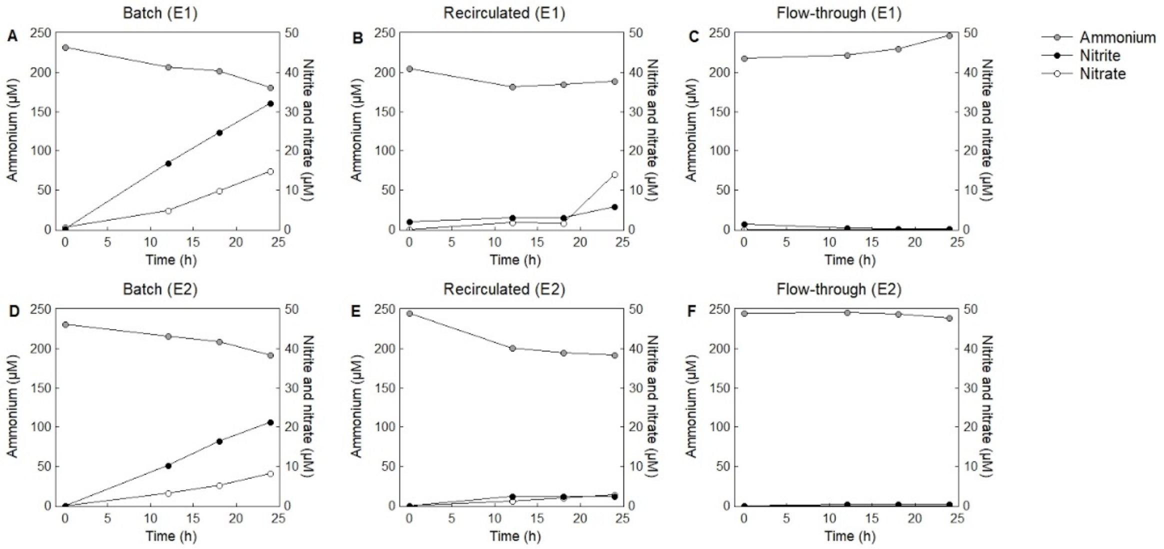

Figure S3. Nitrification activity in batch, recirculated and flow-through incubations of E1 (A-C) and E2 (D-F). Nitrification activity was monitored over the course of the incubation by ammonium consumption, and nitrite and nitrate production. Note that the drop in the ammonium concentration in the recirculated treatment between 0 and 12h was not due to ammonium consumption, but due to dilution by ammonium-free medium that was present in the tubing and filter holder at the beginning of the incubation. Nitrite and nitrate were not detectable in the flow-through incubation due to the strong dilution by the constant medium supply.

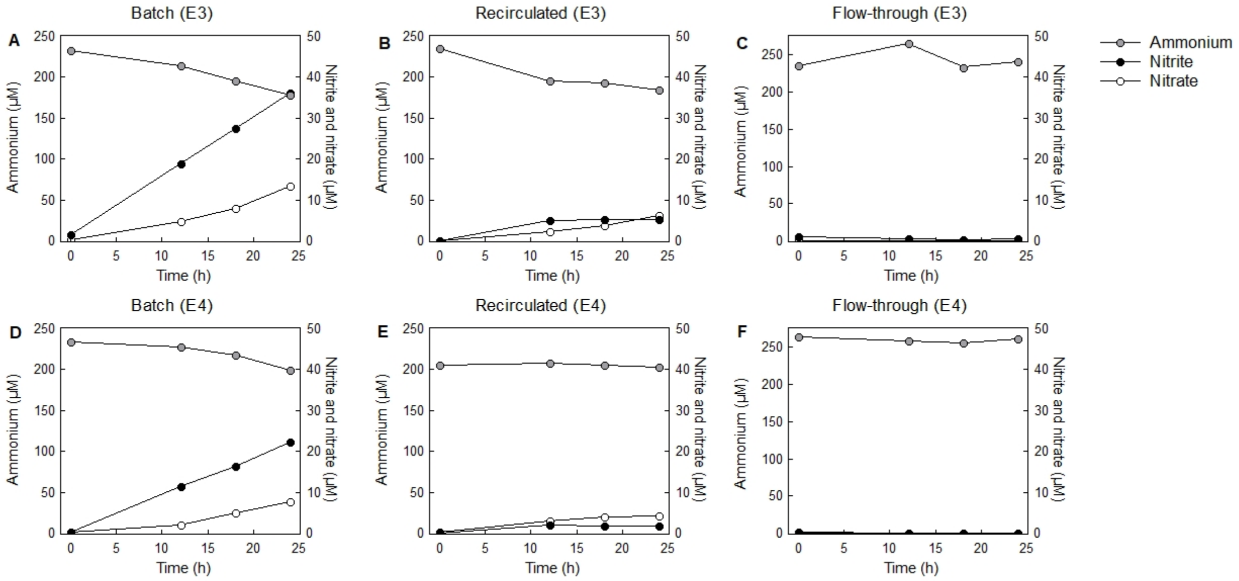

Figure S4. Nitrification activity in batch, recirculated and flow-through incubations of two additional experiments (E3 and E4) to further support reproducibility of the Flow-SIP method. Nitrification activity was monitored over the course of the incubation by ammonium consumption, and nitrite and nitrate production. Note that the drop in the ammonium concentration in the recirculated treatment between 0 and 12h was not due to ammonium consumption, but due to dilution by ammonium-free medium that was present in the tubing and filter holder at the beginning of the incubation. Nitrite and nitrate were not detectable in the flow-through incubation due to the strong dilution by the constant medium supply.

Supplementary tables

Table S1. Material used in flow-through and recirculated approaches. Schemes of the setup of both approaches are given in Figure 1.

Item	Comment	Manufacturer	Article number
Ismatec REGLO ICC digital peristaltic pump; 4-channel, 8-roller		Ismatec	ISM4408
Pump tubing, PharMed® Ismaprene, 1.6 mm inner diameter, 4.8 mm outer diameter, 1.6 mm wall thickness		Ismatec	MF0010
2-stop tubing, PharMed® Ismaprene, 1.65 mm inner diameter		Ismatec	SC0331
In-line stainless steel filter holder, 47 mm		Sartorius	16254
Luer lock connector for filter holders		Sartorius	16881
Whatman® Nuclepore™ Track-Etched Membranes; 47 mm diameter, 0.2 µm pore size, polycarbonate		Whatman	WHA111106
Advantec Grade GC50 Glass Fiber Filters, 47 mm diameter, 0.5 µm pore size	Support filter for E1	Advantec	GC5047MM
GVS Life Sciences Magna™ Nylon Membrane Filters, 47 mm diameter, 0.5 µm pore size	Support filter for E2, Nylon provides a smoother filter surface than the glass fibre support filters used for E1	GVS	1213776
Glass bottle, 1.5 cm inner diameter, 10 cm height, GL25 thread	Medium reservoir for recirculated incubations; custom-made		

Table S2. Test of statistical significance of differences in ^{13}C-enrichment between microbial groups within each approach and differences between approaches within each microbial group. Shown are results of Kruskal-Wallis test and non-parametric multiple comparison test (Dunn's test, see pairwise comparisons column); *P < 0.05, **P < 0.01, ***P < 0.001, n.s. not significant.

		Kruskal-Wallis Test			Dunn's Test, pairwise comparison		
Within approach		χ2	df	P-value	AOB – NOB	NOB – other cells	AOB – other cells
E1	Batch	182.6	2	< 0.001	***	***	***
	Recirculated	134.7	2	< 0.001	***	***	***
	Flow-through	65.1	2	< 0.001	***	n.s.	***
E2	Batch	185.4	2	< 0.001	***	***	***
	Recirculated	193.8	2	< 0.001	***	***	***
	Flow-through	327.6	2	< 0.001	***	n.s.	***

Within microbial group		χ2	df	P-value	Pairwise comparison		
					Batch – Recirc.	Recirc. – Flow-thr.	Batch – Flow-thr.
E1	AOB	157.0	2	< 0.001	***	*	***
	NOB	184.5	2	< 0.001	***	***	***
	Other cells	7.9	2	0.019	*	*	n.s.
E2	AOB	375.0	2	< 0.001	***	***	***
	NOB	98.2	2	< 0.001		***	***
	Other cells	20.3	2	< 0.001	***	***	n.s.
